# Interpreting the Manner of Speech in courts: an overlooked aspect

**DOI:** 10.3389/fpsyg.2023.1209908

**Published:** 2023-12-27

**Authors:** Ran Yi

**Affiliations:** ^1^Australian Human Rights Institute, The University of New South Wales, Sydney, NSW, Australia; ^2^School of Humanities and Languages, The University of New South Wales, Sydney, NSW, Australia

**Keywords:** Manner of Speech, court interpreting, accuracy, language rights, procedural equity

“I put it to you, there is no 10,000 dollars you claimed your mother gave you. You were lying to the court, weren't you?”

—Prosector's Question in Courtroom Cross-Examination

## 1. Introduction

Interpreting is an ancient human activity that dates back to the professional practice performed by “dragoman”, the language interpreter (Pöchhacker, 2022, p. 28; Ruiz Rosendo and Baigorri-Jalón, 2023, p. 1). In modern-day courtrooms, interpreters continue to play an important role in ensuring equity and access to justice. In general settings, interpreters engage in interlingual transfers and bridge across meaning-making systems during interlingual, intercultural, and inter-semiotic oral-gestural exchanges on interpersonal and institutional levels. In institutionalized courtroom interpreting, the meaning of language is particularly nuanced and complex due to a multiplicity of interconnected factors, such as speaker role perceptions, knowledge and experience, and individual linguistic and cultural backgrounds and expectations of institutional culture. For example, a convincing body of literature (see O'Barr, 1982; Woodbury, 1984; Gibbons, 2003; Solan, 2010; Coulthard, 2017; Yi, 2023a) has ascertained that lawyer questions are seldom questions. They are linguistic devices carefully chosen by legal professionals to achieve a strategic aim. In response to lawyer questions, defendants and witnesses may also use linguistic features to express their intent and emotions (Yi, 2023b).

The level of intricacy is further compounded by the presence of an interpreter. In court, when one party has limited proficiency in the official language of the justice system, s/he is entitled to the free assistance of an interpreter. The right to a fair representation through an interpreter is not only a basic human right (see UNICCPR, 1966), but also an integral part of procedural equity (see Civil Code of the People's Republic of China, 2021; Yi, 2023c).

Accuracy of interpreting is paramount to a fair outcome in court (Yi, 2022, 2023d). There are several national documents that provide authoritative explanations of the meaning of accuracy in courtroom settings. This article focuses on three perspectives: (1) practitioner, (2) regulatory, and (3) judiciary. The corresponding representative instruments include (1) the professional code of conduct, (2) recommended standards, and (3) practice notes. In the Australian context, the Australian Institute of Interpreters and Translators Code of Conduct (AUSIT, 2012) defines accuracy in the following way.

“(professional interpreters) should provide accurate renditions of the source utterance or text in the target language. In this case, accurate means (1) optimal and complete; (2) without distortion or omission; (3) preserving the content and intent of the source message or text (p. 5)”.

To achieve accuracy, interpreters should not add to, alter, or omit anything from the content and intent of the source message, ask for clarifications, repetition, or explanation where circumstances permit, and promptly rectify any interpreting mistakes.

The other explanation of accuracy of court interpreting is provided in the Recommended National Standards for Working with Interpreters in Court Tribunals (2022).

“Content and manner are important in hearing room discourse. Interpreters should aim to achieve accuracy of content and manner, including the tone and register of the source language utterances (p. 66).”

The definition above emphasizes that competent and ethical interpreters should not omit information that they consider to be irrelevant. Instead, they will strive to preserve the exact manner, force, and effect in which the original utterances are produced. For example, whether hesitant or confident, the exact tone of the original utterances should be faithfully maintained in the interpreted utterances.

Another example is the Federal Court of Australia's General Practice Note, “Working with Interpreters (GPN-INTERP)”, released on 24 March 2023.^1^ The Note highlights two specific considerations in achieving accuracy expected by the judicial sector: (1) the meaning of interpreting “accurately” and (2) the importance of transferring both the content and the intent of the communication without omission or distortion, as shown below.

“Resulting in the optimal and complete transfer of the meaning from the other language into English and from English into the other language, preserving the content and intent of the communication made in the other language or in English (as the case may be) without omission or distortion and including matters which the interpreter may consider inappropriate or offensive”.

The judicial expectations on accuracy can be dissected into three elements: (1) interpreting everything that has been said in court, including emotionally charged expressions and languages, including curses and hated speech, (2) reproducing what is said and how it is said in court, including the content, manner (through use of fillers, hedges, self-repairs, tone, and intonation), intent (in explicit and implicit form), and (3) applying professional discernment in retaining the optimal and complete transfer to the best of their knowledge and ability.

However, a review of existing literature reveals two main gaps: (1) a definitional clarity of the manner and (2) the importance and difficulties of achieving accuracy in reproducing the manner in court. This short opinion article intends to bridge these gaps in knowledge. It does so by providing a working definition of Manner of Speech and eliciting challenges in reproducing Manner of Speech in court utterances.

## 2. The Manner of Speech in court interpreting

### 2.1. The Manner of Speech: a working definition

The concept of Manner of Speech is multifaceted and fluid. It is, therefore, widely contested and critiqued by scholars for its broad and often inconsistent meaning. One approach to providing some definitional clarity to the term is through a working definition. In her study, she defines this term as “the manner in which speakers express their thoughts and feelings” (Lee, 2011, p. 3). However, her study only looks at speech style features manifested through lexical choice, use of linguistic devices, pronunciation, intonation, stress, pitch, and non-verbal linguistic features, particularly in the Korean language. In this opinion article, I expand her definition by proposing the following working definition in the context of interpreter-mediated court proceedings:

“The manner of speech refers to the manner in which the propositional content of the utterances is produced and presented by the speaker in the context of a courtroom for a particular purpose and reproduced and represented through an interpreter. It can encompass a variety of heterogeneous features. These features include (1) discourse markers, (2) speech style, and (3) other manner-related contextual or interactional cues.”

Manner of Speech serves various functions due to the indexicalities. Theories and practice-informed research have shown that manner-related features are indicative of multiple socio-psychological traits and cognitive processes of the speaker, the hearer, or the interpreter. Theoretical bases in support of this finding include Sperber and Wilson's Relevance Theory, Grice's Manner Maxim, and Searle and Vanderveken's Speech Acts Theory. The manner in which speakers speak is found to be linked to individual linguistic choice, unconscious habits (Olsson, 2008), identity (Fairclough, 2003), and personality (Lakoff, 1979). Based on a review of relevant literature, I also propose an analytical framework that can be further applied, with a particular focus on the Mandarin and English language combination (see Table 1).

**Table 1:** Analytical models for Manner of Speech (Mandarin and English).

**Categories**	**Sub-categories**	**References**
1. Discourse markers	1.1 Acknowledgment markers.	Schiffrin, 1987
	1.2 Politeness markers.	Brown and Levinson, 1987
	1.3 Particle markers.	Heritage, 1990
2. Speech style	2.1 Hesitations.	Wang, 2021
	2.2 Fillers.	Liu and Xiao, 2009; Dayter, 2021
	2.3 Hedges.	Magnifico and Defrancq, 2017; Hu, 2022
	2.4 Self-corrections.	Levelt, 1983
	2.5 Repetitions.	Tree, 1995
3. Other manner-related contextual or interactional cues	3.1 Intonation.	Levis, 2012
	3.2 Tone of voice.	Yip, 2002
	3.3 Register.	Gibbons, 2018

### 2.2. The importance and difficulties in reproducing the Manner of Speech

Existing studies have asserted the importance of preserving markers, speech style, and manner-related features. For example, Lee (2015) reveals that neglect of speech style features can impact the jurors' perceptions of the convincingness of the witness, their evaluation of the testimonies, and their final verdict. Her evidence-based studies point to the fact that inadequate and inaccurate language interpretation in court is detrimental to the counsel's questioning techniques and the credibility of witnesses testimonies, further influencing the outcome of a case.

In practice, reproducing the Manner of Speech intended or implied by the original speaker into the equivalent form with matching force and effect in another language can be rather difficult, particularly in cross-lingual and cross-cultural transfers. In this opinion article, I provide three possible explanations for such difficulties: (1) versatile interpretations of the indexicalities of manner-related features; (2) these features seem less observable, compared with a whole chunk of content-intensive speech marked by legal arguments, facts, and sources of law in courtroom examinations; and (3) manner-related features seem to be less substantive to the case. To put it simply for general readers, Manner of Speech can mean different things to different people and members of socio-cultural groups and language communities with varied expectations of institutional culture and traditions.

However, this opinion article establishes counter-claims: (1) the Manner of Speech is equally important as the propositional content of the utterances, as reflected in professional guidelines, interpreting protocols, and judicial practice notes reviewed in earlier part of this article; (2) not rendering the Manner of Speech may have implications for the judicial outcome of the case in many ways, as found in previous studies (see Hale, 2004; Lee, 2009, 2011, 2015; Stern and Liu, 2019; Liu, 2020; Hale et al., 2022; Yi, 2024); and (3) Manner of Speech is observable, it is manifested through the use of multiple devices, including acknowledgment markers (e.g., well/好的), politeness markers (e.g., please/请), and rapport building devices and contextual or interactional cues. Therefore, it is very important to (1) increase the awareness of the Manner of Speech in interpreter-mediated court interactions, (2) improve inter-professional understanding and collaboration rooted in mutual purpose and shared expectations, and (3) develop manner-related pedagogical resources in interpreter education.

## 3. Conclusion

This short opinion article is intended as a position paper for general readers. There have been several studies on question types (Liu, 2020), reported speech (Cheung, 2012, 2014, 2017, 2018) and speech style features (Lee, 2009, 2011) and the implications for procedural fairness and judicial outcomes in interpreter-mediated courtroom interactions. However, little has been explored about the concept of the Manner of Speech. Written in a non-specialized manner and in plain language, this article strives to make a point by shedding light on a long-overlooked aspect in existing studies. Due to its limited scope, this short article only provides a general overview of key issues, theoretical approaches, analytical models, and factors related to the under-explored aspect of the understanding of accuracy in interpreter-mediated court encounters. As shown in the professional code of conduct, recommended standards, and court's practice note, it is important to emphasize that the accuracy of interpreting in court is not merely about reproducing what is said but also rendering how it is said to the best of the interpreter's knowledge. To faithfully reproduce the manner in which the propositional content is expressed, a competent interpreter can resort to the pragmalinguistic approach and strive for equivalent effect and force in the interpretations of utterances during courtroom examinations.

Echoing Morris (1995) on the dilemmas of court interpreting, this article claims that an adequate and accurate language interpretation in court is not merely a rights issue pertaining to procedural equity and social justice but also a moral imperative linking to professionally ethical conduct. Therefore, this opinion article calls for doing justice to the Manner of Speech in future research studies, professional and pedagogical practices. Several directions for future studies include (1) further development of language-specific conceptual models, (2) in-depth analyses of specific manner-related features, and (3) experimental research examining cognitive or contextual factors that impact the reproduction of manner-related features in simulated courtroom settings.

## Author contributions

The author confirms being the sole contributor of this work and has approved it for publication.

## Footnotes

^1^See Working with Interpreters (GPN-INTERP). Retrieved from: https://www.fedcourt.gov.au/law-and-practice/practice-documents/practice-notes/gpn-interpret (accessed May 29, 2023).

## References

[B1] AUSIT (2012). Code of Conduct. Retrieved from: https://ausit.org/wp-content/uploads/2020/02/Code_Of_Ethics_Full.pdf (accessed October 31, 2022).

[B2] Brown P. Levinson S. C. (1987). Politeness: Some Universals in Language Usage. Cambridge: Cambridge University Press.

[B44] Cheung A. K. (2012). The use of reported speech by court interpreters in Hong Kong. Interpreting 14, 73–91. 10.1075/intp.14.1.04che

[B3] Cheung A. K. (2014). The use of reported speech and the perceived neutrality of court interpreters. Interpreting 16, 191–208. 10.1075/intp.16.2.03che

[B4] Cheung A. K. (2017). Non-renditions in court interpreting: a corpus-based study. Babel 63, 174–199. 10.1075/babel.63.2.02che

[B5] Cheung A. K. (2018). Non-renditions and the court interpreter's perceived impartiality: a role-play study. Interpreting 20, 232–258. 10.1075/intp.00011.che

[B6] Civil Code of the People's Republic of China (2021). Retrieved from: https://english.lawsregulations/202012/31/content_WS5fedad98c6d0f72576943005.html (accessed April 21, 2023).

[B7] Coulthard M. (2017). An Introduction to Forensic Linguistics: Language in Evidence. London: Routledge.

[B8] Dayter D. (2021). Variation in non-fluencies in a corpus of simultaneous interpreting vs. non-interpreted English. Perspectives. 29, 489–506. 10.1080/0907676X.2020.1718170

[B9] Fairclough N. (2003). Analysing Discourse: Textual Analysis for Social Research. London: Routledge.

[B10] Gibbons A. (2018). Contemporary Stylistics: Language, Cognition, Interpretation. Edinburgh: Edinburgh University Press.

[B11] Gibbons J. (2003). Forensic Linguistics: An Introduction to Language in the Justice System. New Jersey, NJ: Blackwell.

[B12] Hale S. (2004). The Discourse of Court Interpreting: Discourse Practices of the Law, the Witness, and the Interpreter. John Benjamins.

[B13] Hale S. Goodman-Delahunty J. Martschuk N. Doherty S. (2022). The effects of mode on interpreting performance in a simulated police interview. Transl. Interpret. Stud. 17, 264–286. 10.1075/tis.19081.hal

[B14] Heritage J. (1990). Intention, meaning and strategy: Observations on constraints on interaction analysis. Res. Lang. Soc. Interact. 24, 311–332.

[B15] Hu J. (2022). Hedges in Chinese-English Conference Interpreting. Cham: Springer.

[B16] Lakoff R. T. (1979). “Stylistic strategies within a grammar of style,” in Language, Sex, and Gender, eds J. Orasanu, M. Slater, and L. L. Adler (New York, NY: New York Academy of Sciences), 53–78.

[B17] Lee J. (2009). Interpreting inexplicit language during courtroom examination. Appl. Linguist. 30, 93–114. 10.1093/applin/amn050

[B18] Lee J. (2011). Translatability of speech style in court interpreting. Int. J. Speech Lang. Law 18, 1–33. 10.1558/ijsll.v18i1.1

[B19] Lee J. (2015). Evaluation of court interpreting: a case study of metadiscourse in interpreter-mediated expert witness examinations. Interpreting 17, 167–194. 10.1075/intp.17.2.02lee

[B20] Levelt W. J. M. (1983). Monitoring and self-repair in speech. Cognition. 14, 41–104. 10.1016/0010-0277(83)90026-4 6685011

[B21] Levis J. M. (2012). Supreasegmentals: Intonation. The Encyclopedia of Applied Linguistics. Wiley Online Library. 10.1002/9781405198431.wbeal1124

[B22] Liu B. Xiao E. (2009). “Chinese discourse markers in oral speech of mainland Mandarin speakers,” in Proceedings of the 21st North American conference on Chinese linguistics (NACCL-21), Vol. 2 (Smithfied, RI: Bryant University), 358–374.

[B23] Liu X. (2020). Pragmalinguistic challenges for trainee interpreters in achieving accuracy: an analysis of questions and their interpretation in five cross-examinations. Interpreting 22, 87–116. 10.1075/intp.00035.liu

[B24] Magnifico C. Defrancq B. (2017). Hedges in conference interpreting: The role of gender. Interpreting. 19, 21-46. 10.1075/intp.19.1.02mag

[B25] Morris R. (1995). The moral dilemmas of court interpreting. Translator 1, 25–46. 10.1080/13556509.1995.10798948

[B26] O'Barr W. M. (1982). Linguistic Evidence: Language, Power, and Strategy in the Courtroom. Cambridge, MA: Academic Press.

[B27] Olsson J. (2008). Forensic Linguistics. London: Continuum.

[B28] Pöchhacker F. (2022). Introducing Interpreting Studies. London: Routledge.

[B29] Recommended National Standards for Working with Interpreters in Court Tribunals 2022 Judicial Council on Cultural Diversity. (2022). Recommended National Standards for Working with Interpreters in Court and Tribunals. Available online at: https://jccd.org.au/wp-content/uploads/2022/05/JCDD-Recommended-National-Standards-for-Working-with-Interpreters-in-Courts-and-Tribunals-second-edition.pdf

[B30] Ruiz Rosendo L. Baigorri-Jalón J. (2023). Towards an Atlas of the History of Interpreting: Voices From Around the World. Amsterdam: John Benjamins.

[B31] Schiffrin D. (1987). Discourse Markers. Cambridge: Cambridge University Press.

[B32] Solan L. M. (2010). The Language of Judges. Chicago, IL: University of Chicago Chicago Press.

[B45] Stern L. Liu X. (2019). See you in court: how do Australian institutions train legal interpreters? Interpr. Transl. Train. 13, 361–389. 10.1080/1750399X.2019.1611012

[B33] Tree J. E. F. (1995). The effects of false starts and repetitions on the processing of subsequent words in spontaneous speech. J. Mem. Lang. 34, 709–738. 10.1006/jmla.1995.1032

[B34] UNICCPR (1966). The United Nations International Covenant on Civil and Political Rights. Retrieved from: https://treaties.un.org/doc/publication/unts/volume%20999/volume-999-i-14668-english.pdf (accessed April 21, 2023).

[B35] Wang Y. B. (2021). A study on the use of hesitation markers in varied-level EFL learners' L2 speaking process. Open J. Mod. Linguist. 11, 823–840. 10.4236/ojml.2021.115063

[B36] Woodbury H. (1984). The strategic use of questions in court. Semiotica 48, 197–228. 10.1515/semi.1984.48.3-4.197

[B37] Yi R. (2022). Does style matter in remote interpreting: a survey study of professional court interpreters in Australia. Int. J. Transl. Interpret. Stud. 2, 48–59. 10.32996/ijtis.2022.2.1.7

[B38] Yi R. (2023a). Preparing future court interpreters how are questions phrased in virtual court settings? Lingua Legis. 30, 23–43. 10.13140/RG.2.2.33645.97766

[B39] Yi R. (2023b). Human interpreters in virtual courts: a review of technology-enabled remote settings in Australia. J. Digit. Technol. Law. 1, 712–724. 10.21202/jdtl.2023.31

[B40] Yi R. (2023c). The promise of linguistic equity for migrants in Australian courtrooms: a cross-disciplinary perspective. Aust. J. Hum. Rights 29, 174–180. 10.1080/1323238X.2023.2232171

[B41] Yi R. (2023d). Assessing the manner of speech in Australian courts: a study of Chinese-English professional interpreters in remote settings. Int. J. Public Service Interpret. Transl. 10, 35–47. 10.37536/FITISPos-IJ.2023.10.1.339

[B42] Yi R. (2024). Justice under microscope: analysing mandarin chinese markers in virtual courtroom discourse. Discourse Stud. 10.1177/14614456231197045

[B43] Yip M. (2002). Tone. Cambridge: Cambridge University Press.

